# Structure and Biomechanics during Xylem Vessel Transdifferentiation in *Arabidopsis thaliana*

**DOI:** 10.3390/plants9121715

**Published:** 2020-12-05

**Authors:** Eleftheria Roumeli, Leah Ginsberg, Robin McDonald, Giada Spigolon, Rodinde Hendrickx, Misato Ohtani, Taku Demura, Guruswami Ravichandran, Chiara Daraio

**Affiliations:** 1 Department of Materials Science and Engineering, University of Washington, Seattle, WA 98195, USA; 2 Division of Engineering and Applied Science, California Institute of Technology, Pasadena, CA 91125, USA; lginsber@caltech.edu (L.G.); robinmcd@caltech.edu (R.M.); rodindehendrickx@hotmail.com (R.H.); ravi@caltech.edu (G.R.); daraio@caltech.edu (C.D.); 3 Biological Imaging Facility, California Institute of Technology, Pasadena, CA 91125, USA; giadas@caltech.edu; 4 Department of Integrated Biosciences, Graduate School of Frontier Sciences, The University of Tokyo, Kashiwa 277-8562, Japan; misato@edu.k.u-tokyo.ac.jp; 5 Graduate School of Biological Sciences, Nara Institute of Science and Technology, Ikoma 630-0192, Japan; demura@bs.naist.jp

**Keywords:** plant biomechanics, turgor pressure, micro-compression, AFM, *Arabidopsis thaliana*, differentiation

## Abstract

Individual plant cells are the building blocks for all plantae and artificially constructed plant biomaterials, like biocomposites. Secondary cell walls (SCWs) are a key component for mediating mechanical strength and stiffness in both living vascular plants and biocomposite materials. In this paper, we study the structure and biomechanics of cultured plant cells during the cellular developmental stages associated with SCW formation. We use a model culture system that induces transdifferentiation of *Arabidopsis thaliana* cells to xylem vessel elements, upon treatment with dexamethasone (DEX). We group the transdifferentiation process into three distinct stages, based on morphological observations of the cell walls. The first stage includes cells with only a primary cell wall (PCW), the second covers cells that have formed a SCW, and the third stage includes cells with a ruptured tonoplast and partially or fully degraded PCW. We adopt a multi-scale approach to study the mechanical properties of cells in these three stages. We perform large-scale indentations with a micro-compression system in three different osmotic conditions. Atomic force microscopy (AFM) nanoscale indentations in water allow us to isolate the cell wall response. We propose a spring-based model to deconvolve the competing stiffness contributions from turgor pressure, PCW, SCW and cytoplasm in the stiffness of differentiating cells. Prior to triggering differentiation, cells in hypotonic pressure conditions are significantly stiffer than cells in isotonic or hypertonic conditions, highlighting the dominant role of turgor pressure. Plasmolyzed cells with a SCW reach similar levels of stiffness as cells with maximum turgor pressure. The stiffness of the PCW in all of these conditions is lower than the stiffness of the fully-formed SCW. Our results provide the first experimental characterization of the mechanics of SCW formation at single cell level.

## 1. Introduction

Plantae and plant-based materials are specialized conglomerates of plant cells. Therefore, studying the mechanical properties of single cells and resolving further sub-cellular contributions provides a basis for further analysis of the heterogeneous tissue and plant-level biomechanics. In vascular plant tissues, the micro-structure and composition of secondary cell wall (SCW) governs, to a large extent, the mechanical properties of the entire tissue [1,2]. Thus, it is of paramount importance to investigate the mechanical properties of the SCW, especially during the initial stages of formation, which has not been explored to date.

Plant cells have two key structural elements that collectively govern their mechanical properties: the cell wall and the cytoskeleton. The key structural component of the cell wall is cellulose, which has a Young’s modulus (E=110–220 GPa) comparable to that of high performance engineering materials like carbon fiber or steel [3]. Cellulose is immersed in an amorphous matrix of softer biopolymers, hemicellulose, pectin, proteins and lignin, giving rise to a complex heterogeneous multilayered cell wall structure [4]. The support provided to plant cells by the cell wall allows them to hold water at high pressures (p=0.3–1.0 MPa), mainly through swelling of the vacuole [5]. This phenomenon in plants is known as turgor pressure, and it is essential to the structural integrity and rigidity of the cell. Additional structural support is provided to the cell by the cytoskeleton, largely through actin filaments (E=1.0–4.0 kPa) and microtubules (E=1.1–1.3 GPa) [6,7,8].

Recent advances in instrumentation are the impetus for the resurgence in research focused on the mechanics of cell growth [9,10,11]. Experimental methods, protocols and mechanical models of plant cells vary, contributing to results that span orders of magnitude [9]. Still, newly designed experiments have the potential to achieve an unprecedented spatial resolution and therefore to decouple the mechanical contributions from each structural element of a cell in the overall mechanical performance of plant cells and tissues. For example, Routier-Kierzkowska et al. designed an experimental apparatus which they termed as cellular force microscope and used it to create stiffness maps of onion epidermis peels [12]. The results of this new experiment in combination with finite-element simulations revealed that turgor pressure caused the observed stiffening on top of inflated cells. Braybrook and Peaucelle performed AFM indentations on plasmolyzed Arabidopsis tissues, which ensured the isolation of the response of the cell wall from any contribution due to turgor pressure [13]. By measuring the response from the plasmolyzed tissue, they were able to demonstrate that auxin leads to wall acidification in preparation for cell expansion.

This cell wall loosening behavior in preparation for elongation was first observed by Radotić et al. who performed AFM indentation measurements on suspension-cultured Arabidopsis cells and observed that the cell wall stiffness at the beginning and end of cell growth was almost an order of magnitude lower than during the exponential growth phase [14]. The mechanism behind this cell wall loosening in preparation for elongation has been explained by Cosgrove in a subsequent review [4].

Even though many are working towards a defined micromechanical model, the exact contribution of the cell wall(s) and cytoplasmic components on the effective stiffness of the system during growth and differentiation remains elusive. Using a novel protocol to characterize the rheology of isolated Arabidopsis protoplasts, Durand-Smet et al. found that the elastic modulus of the protoplast was about three orders of magnitude lower than plant cells with a cell wall [8]. In the same work, plant cells were treated with a microtubule destabilizing drug, which reduced the elastic modulus of the protoplast to half of its original value, demonstrating that MTs contribute to the overall stiffness of the cell. Sampathkumar et al. used live-imaging of *Arabidopsis thaliana* (Arabidopsis) plants, particularly in epithelial cells, and a mechanistic model to find that there is a direct correlation between microtubule (MT) organization and geometry-derived mechanical stresses [15]. Apparently, the maximum stress in the cell wall is found in areas with highest cellulose concentration, which is driven by the MTs in the cytoplasm. Taken together, the results of Durand-Smet et al. and Sampathkumar et al. show that MTs contribute to the overall stiffness of cells intrinsically, and through an interaction with the cell wall. Here, in order to understand the mechanical contributions of the subcellular components, like the cell wall(s) and cytoplasm, throughout the transdifferentiation process, we propose a robust multi-scale mechanics assay that includes nano-indentation to capture cell wall properties, chemical treatments to control osmotic conditions and micro-indentation to evaluate global cell properties.

We choose to focus on xylem vessel element differentiation, which is one of the most extensively used systems to study SCW development and thickening [16,17]. Xylem vessel elements develop a precisely patterned SCW beneath the primary cell wall (PCW) giving rise to an entangled multilayered heterostructure. The deposition of SCW in xylem vessel elements is intricately linked to programmed cell death (PCD), and both processes are happening concurrently during differentiation [18]. Therefore, quantifying the mechanical contributions of the cell wall(s) and cytoplasm during differentiation of xylem vessel elements is a convoluted problem, and one that has not yet been solved. Our multi-scale biomechanical assay is designed to capture mechanical contributions from the PCW, the SCW, their potential coupled effects, as well as the cytoskeleton at various turgor pressures and osmotic conditions.

Early in vitro SCW induction systems for *Zinnia elegans* facilitated physiological, biochemical, and molecular studies that elucidated the tracheary element (TE) differentiation mechanism [19,20,21]. The Demura group introduced the post-translational induction system of VASCULAR-RELATED NAC-DOMAIN7 (VND7) genes which induces transdifferentiation of various types of plant cells into xylem vessel elements upon treatment with a glucocorticoid, such as dexamethasone (DEX) [16,17]. The induction system has been demonstrated successfully in Arabidopsis plants and cell cultures, as well as *Populus tremula x tremuloides* plantlets, and *Nicotiana tabacum* cell cultures [16]. The system causes the activation of transcriptional activity of VND7 to induce ectopic transdifferentiation of Arabidopsis cultured cells into protoxylem vessel-like cells [16].

In this study, we use the VND7 system in Arabidopsis suspension-culture cells because it is a robust model with a high efficiency in transdifferentiation and uniformity in cell culture. To decouple the effects of cell wall stress, cytoskeleton rearrangement, and turgor pressure on observed cell stiffness, we test transgenic Arabidopsis cells in an extensive multi-scale biomechanical assay. To validate the cell wall stiffness decoupled from turgor pressure, we perform AFM indentations [22]. We propose a mechanistic spring model to represent the stiffness of the cell in compression, which allows the decoupling of stiffness contributions from the cell wall(s) and cytoplasm.

## 2. Results and Discussion

### 2.1. Morphological Observations of the VND7-Inducible Arabidopsis Cells

**Figure 1:**
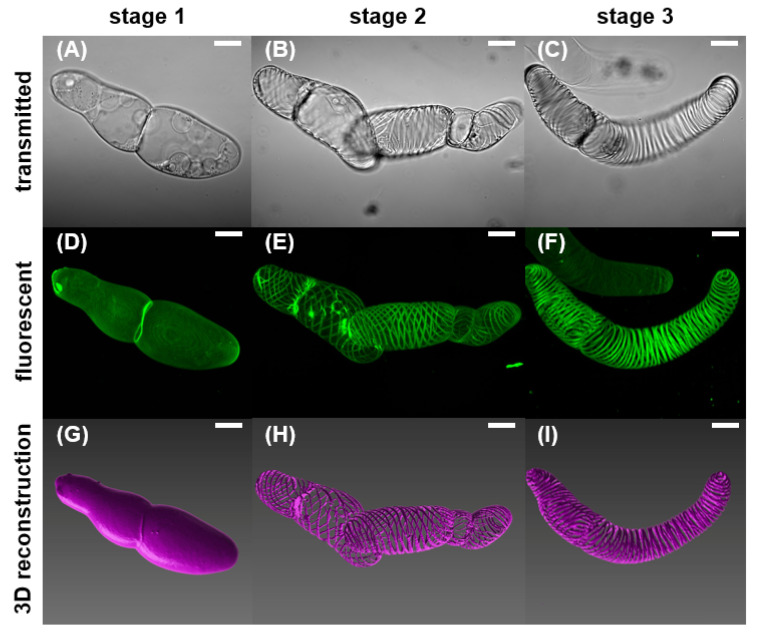
Transmitted, fluorescent, and 3D reconstructions of confocal images of the elongated VND7-inducible cells in the 3 stages of transdifferentiation. (**A**,**D**,**G**) Living cells which have only developed a PCW are identified as stage 1. The PCW is under stress from the internal turgor pressure. (**B**,**E**,**H**) Cells in stage 2 have both a PCW and the beginnings of a SCW. In this stage, the PCW has possibly begun hydrolyzing, and thin spiral bundles of SCW can be distinguished. The spiral patterning is characteristic of protoxylem vessels. (**C**,**F**,**I**) In stage 3, SCW thickening is observed; PCD has progressed; the tonoplast has ruptured and cytoplasmic contents have been removed from the cell. All scale bars are 20 μm.

**Figure A1:**
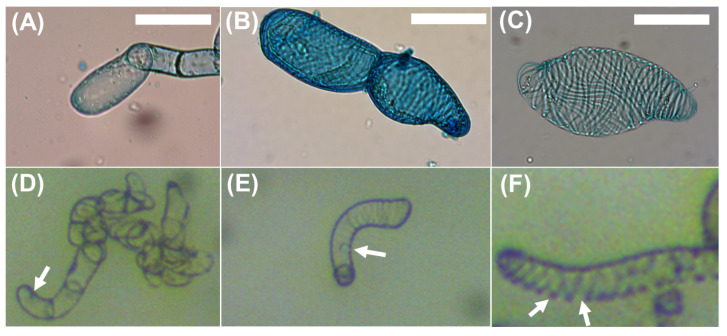
(**A**–**C**) Optical microscopy images and (**D**–**F**) images from the micro-mechanical testing device to demonstrate cell classification process. (**A**,**D**) Living cells in stage 1 of transdifferentiation (only PCW observed, arrow points to cell nucleus). (**B**,**E**) Living cells in stage 2 of transdifferentiation (PCW and SCW are both present, cytoplasm is still inside the cell, arrow points to cell nucleus); (**C**,**F**) Living cells in stage 3 of transdifferentiation (SCW and partially perforated PCW, cytoplasmic contents almost entirely removed, arrow points to locations where PCW is degraded). All scale bars are 20 μm.

The VND7-inducible Arabidopsis cells were stained and observed under a laser scanning confocal microscope at various stages of their differentiation. We document that transdifferentiation of VND7-inducible cells follows the same general stages as TE differentiation seen in other plant systems [19,20,21]. Common morphological observations during differentiation of TEs in *Zinnia elegans*, *Populus deltoides* and Arabidopsis, in the order that they occur, are: (i) the differentiating cell expands and becomes highly vacuolated and the nucleus is confined, pushing against the cell wall and marking the initiation of PCD; (ii) the cytoskeleton rearranges as the cell produces vesicles which have been associated with substance exchange between the cytoplasm and cell wall for SCW deposition; (iii) tonoplast ruptures as SCW is deposited and starts thickening; (iv) following SCW thickening, in planta, PCW perforation is observed [18,23,24,25]. From the transmitted and confocal fluorescent images, as well as optical microscopy images (see Figure A1A–C), we can robustly identify three distinct stages of cell transdifferentiation based on the cell wall(s) and cytoplasm, as presented in Figure 1A–F. Specifically, in the VND7 system we study in this work, we classify the stages as follows: (i) Stage 0: cells prior to induction of transdifferentiation (prior to adding DEX in the solution), having only a PCW developed and visible. (ii) Stage 1: cells having been induced (exposed to DEX for a minimum of 24 h) and having only a PCW developed. (iii) Stage 2: cells having been induced (exposed to DEX for a minimum of 24 h) and having an intact PCW and a SCW developed. The cytoplasmic contents of these cells are visible and still inside the cell. In our observations, cytoplasm retraction and detachment from the cell wall happens soon after SCW deposition. (iv) Stage 3: cells having been induced (exposed to DEX for a minimum of 48 h) having a thickened SCW, and partially perforated PCW. With or without perforated PCW, in stage 3 the majority of cytoplasmic contents are removed from the cells (indicating the tonoplast rupture). Using the confocal fluorescent images, we compile three-dimensional reconstructions for each identifiable stage of transdifferentiation, presented in Figure 1G–I, which allow the evaluation of the PCW thickness, as well as visualization of the bundled SCW thickenings.

**Figure 2:**
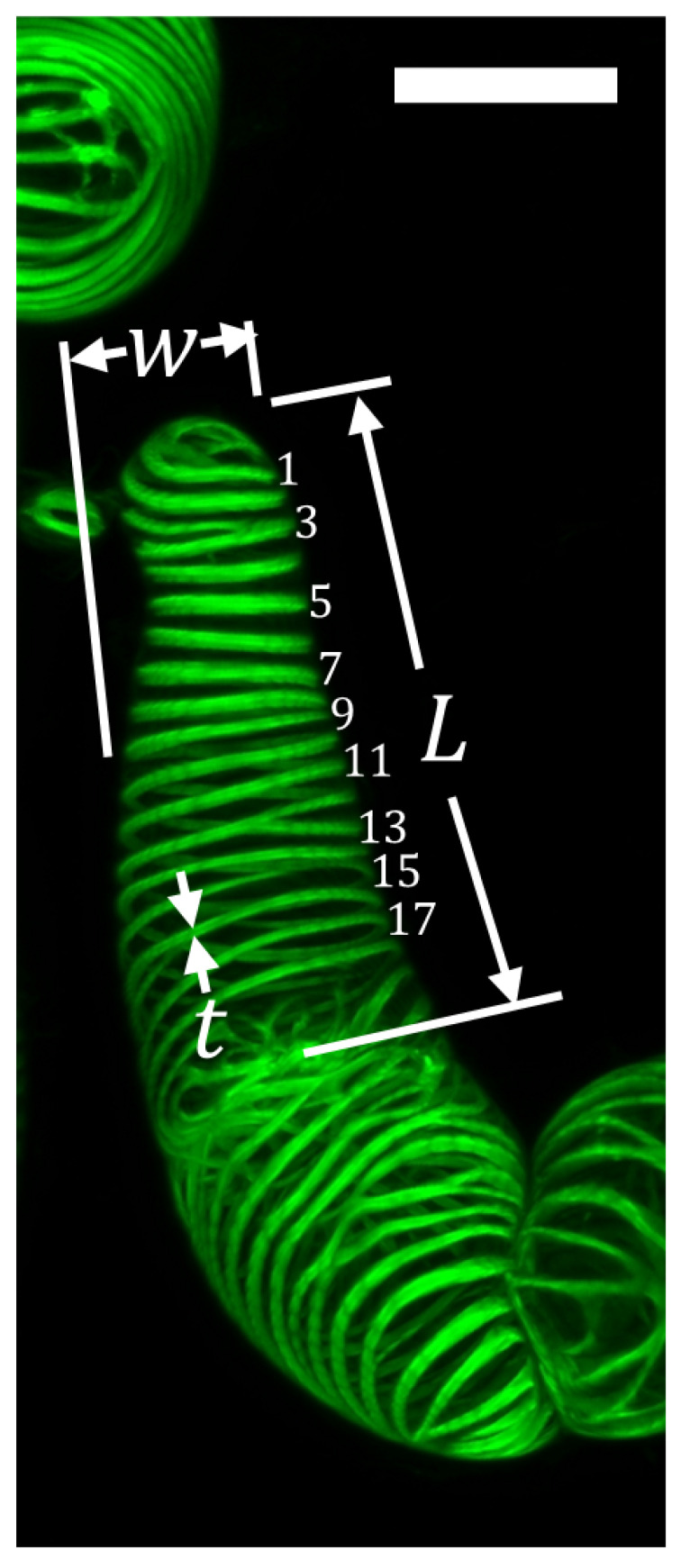
Illustration of measured principal dimensions and feature sizes of elongated VND7-inducible cells from confocal fluorescent microscopy image. Scale bar is 20 μm.

From confocal and additional light microscopy images (data not presented here), we discern two equally represented shapes in the cell population, based on their aspect ratio: rounded and elongated. Elongated cells have a mean aspect ratio of approximately 2:1, whereas rounded cells have a mean aspect ratio of approximately 1:1. Even though the microscopy images denote that approximately half of the population of cells are rounded, and half are elongated, we observe that rounded cells tend to be tightly clustered, while elongated cells are found more likely in an isolated state or located on the edges of large clusters. For that reason, all mechanical data in the following sections are measured from elongated cells, and we will focus on the morphology of those cells in this section. For all dimensions and feature sizes of both rounded and elongated cells, see Table A1 and Table A2. The principal dimensions and feature sizes of elongated cells, along with an illustrative example are shown in Table 1 and Figure 2, respectively.

**Figure A2:**
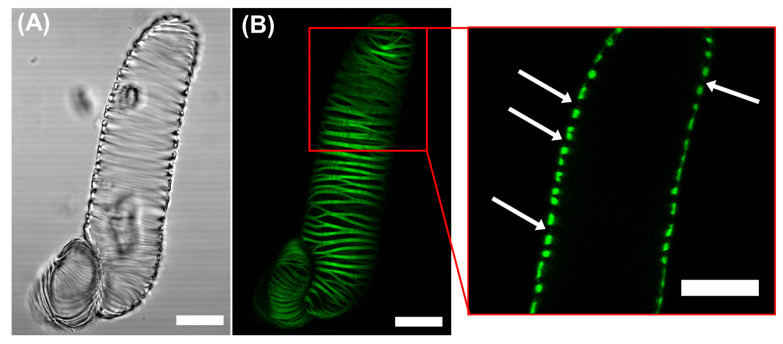
Transmitted and fluorescent images of an elongated VND7-inducible cell in stage 3 of transdifferentiation with visible hydrolyzed PCW. (**A**) Transmitted light image of the cell showing loss of most cytoplasmic contents. (**B**) Fluorescent image of cell highlighting location of SCW bundles. (inset) Single slice of fluorescent confocal image with arrows pointing to gaps in the cell wall, showing that we indeed have loss of PCW material in some locations. All scale bars 20 μm.

Confocal imaging reveals a PCW in stage 1 (thickness 580 ± 10 nm (Mean ± Standard Error)), while bundles of SCW in spiral patterns are observed in stages 2 and 3 of transdifferentiation. In stage 2, the early SCW bundles are deposited, and the cell begins to undergo PCD. In stage 3, as PCD progresses, the SCW bundles are thickened further, the tonoplast ruptures, contents of the cytoplasm are degraded and removed from the cell, and the PCW is at least partially hydrolyzed [2]. (See Figure A2 for partial PCW perforation at stage 3). During the last stage of differentiation, the SCW bundles thicken by approximately 40%. The bundle density does not change notably between stages 2 and 3.

### 2.2. Biomechanics of Differentiating VND7-Inducible Arabidopsis Cells

All reported mechanical data in the following sections are measured from elongated cells in an isolated state. In the mechanical testing we add stage 0 to the differentiation stages, which describes transgenic cells prior to exposure to DEX, reflecting cells in their state before transdifferentiation is induced. There is no observable difference between stages 0 and 1 using the confocal or light microscope, but it has been reported that from stage 0 to 1, the MT and actin filaments reorganize the cytoplasmic fibrillar network into a bundled conformation that will later guide the spiral SCW patterning [26,27]. In fact, the MT-rearrangement in particular has been visualized in the VND7-inducible system and has been reported in literature [28].

**Figure 3:**
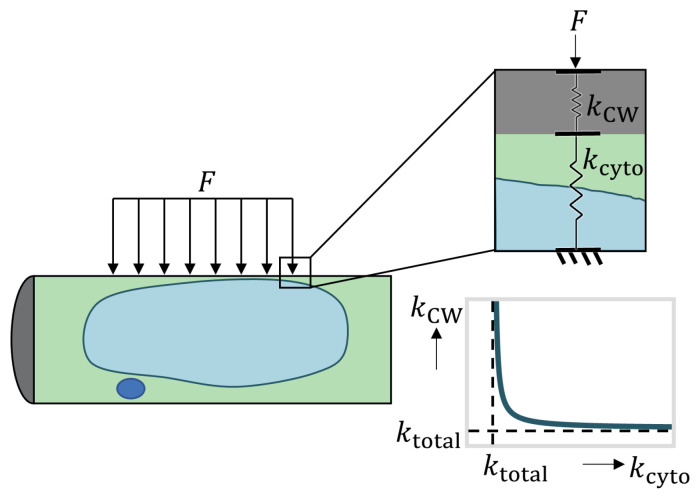
Proposed series spring model to analyze cell stiffness from micro-compression testing. The total stiffness measured by the micro-compression tool is the equivalent stiffness for the two springs in series, given by Equation (1), and it must be less than the intrinsic stiffness of either constituent spring in the series. For a given overall stiffness, $k_{\mathrm{total}}$, the included plot illustrates the relationship between the stiffness of the two springs, $k_{\mathrm{CW}}$ and $k_{\mathrm{cyto}}$.

The elongated cells were compressed using a micro-compression tool that covered most of their top surface area. We propose a spring model to describe the overall cell stiffness, as pictured in Figure 3. The pictured model has two springs in series, one which represents the stiffness of the cell wall, and the other represents the stiffness of the cytoplasm. The combined stiffness of these two springs is given by the following equation:

(1) $$k_{\mathrm{total}}=\frac{k_{\mathrm{CW}}k_{\mathrm{cyto}}}{k_{\mathrm{CW}}+k_{\mathrm{cyto}}}$$

A direct result of using this model is that the overall stiffness must be less than the stiffness of either constituent springs. In the following sections we will use this proposed model to deconvolute the stiffness contributions from the cell wall(s) and cytoplasm. The result is a ranking of the stiffness contributions from the cell wall(s) and cytoplasm in each stage of differentiation. It is important to note that this ranking depends on the validity of the assumptions outlined in the following paragraph.

**Figure A3:**
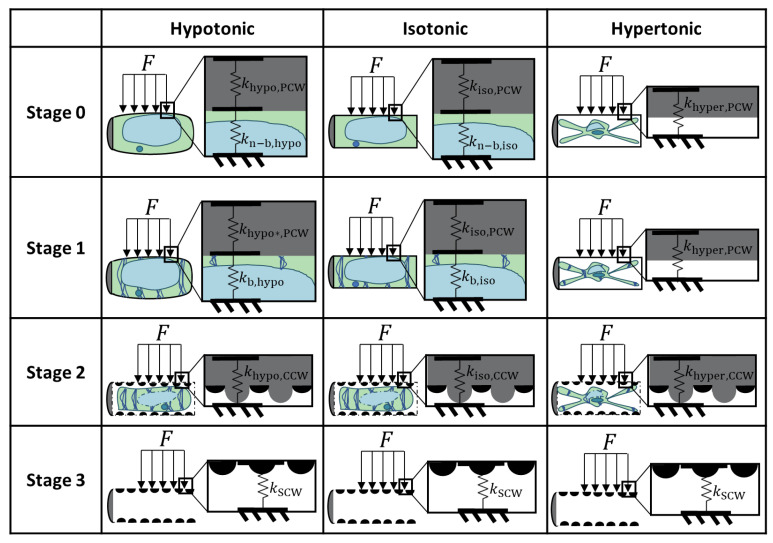
Spring models for all treatments/stages.

In using this simplified model, we assume that the cell wall and cytoplasm behave as linear elastic materials at small indentation depths, and we ignore any nonlinear effects like adhesion, viscosity, or plasticity. For shallow indentations, the effects from stress at the boundaries of the cell also become negligible [29]. This simplified one-dimensional model allows us to quantify the relative stiffness contributions of each component. Stiffness is not an intrinsic material property, like Young’s modulus which is independent of the shape and dimensions of the material, since by definition stiffness is a function of both the material and it’s geometry. To relate spring stiffness to Young’s modulus would require a three-dimensional model which captures the structural mechanics of a pressurized cell with a heterogeneous membrane undergoing large deformations. Due to the lack of such a model in the current literature, we adopt this one-dimensional spring model, which allows us to decouple the relative contributions from the cytoplasm and cell wall(s), although we cannot yet directly obtain intrinsic material properties for either component. See Figure A3 for the specific spring models used to represent cells in each stage of transdifferentiation, and in each solution with different osmolarity.

**Figure 4:**
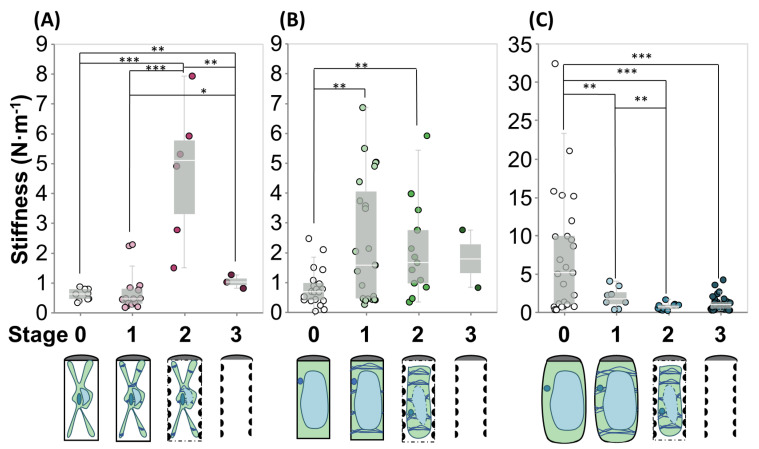
Panel showing the stiffness in 4 stages of transdifferentation in 3 types of solutions with different osmolarity: (**A**) sorbitol; (**B**) growth medium; (**C**) water. Note the difference in scale on the y-axis in (**C**) from the extreme stiffness of cells in hypotonic conditions before induction of differentiation. Bottom line graphically represents the morphology of the cells in each condition and stage. Stars indicate significant differences in distribution according to the nonparametric Kolmogorov-Smirnov test. * *p* < 0.1, **p < 0.05, *** *p* < 0.01. (Data shown correspond to 2 < n < 35. Each indentation test on an individual cell is represented by a point in the plot.)

**Figure A4:**
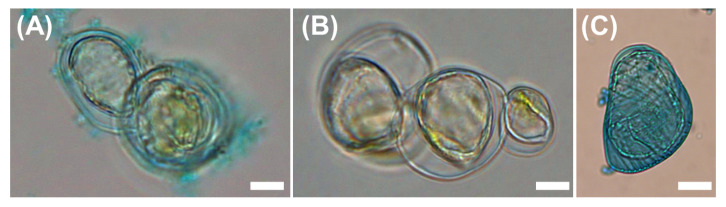
Transmitted light images of VND7-inducible cells immersed in sorbitol solution for 1 min visualizing the immediate effects of plasmolyzing conditions. Cytoplasm retraction upon plasmolysis is observed for cells at (**A**) stage 0, (**B**) stage 1, (**C**) stage 2. All scale bars 20 μm.

**Figure A5:**
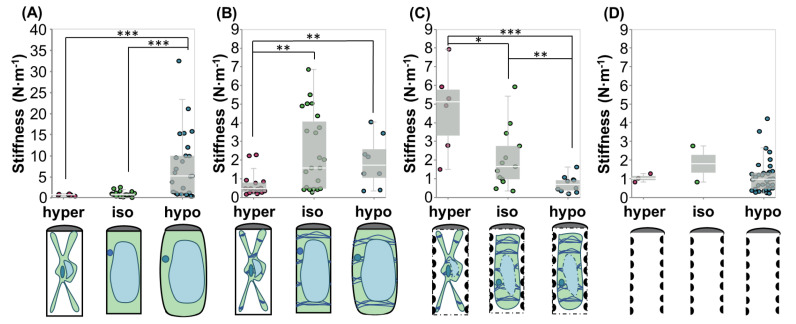
Panel showing stiffness in hypertonic, isotonic, and hypotonic solutions in 4 stages of transdifferentiation: (**A**) stage 0; (**B**) stage 1; (**C**) stage 2; (**D**) stage 3. Stars indicate significant differences in distribution according to a Kolmogorov-Smirnov test. * *p* < 0.1, **p < 0.05, *** *p* < 0.01.

For the micro-indentation tests, we extract cells from their normal growth conditions (in growth media) at different time points before and after exposure to DEX, from 24 h to several days, evaluate their morphology in an optical microscope (see Figure A1A–C) and identify their stage of transdifferentiation. Testing of cells at different time points after exposure to DEX allows us to capture them at each of the four identified differentiation stages. We confirm the stage of differentiation and cell morphology through the optical microscope embedded in the micro-mechanical testing system (see Figure A1D–F). After the extraction from normal growth conditions, we treat the cells in three different osmotic conditions and maintain them during the mechanical testing, which is conducted in solution. We refer to the testing conditions as hypertonic, when cells are in sorbitol, isotonic, when cells are in growth media, and hypotonic, when cells are in deionized water. See Table A3 for details on the calculation of the osmotic pressure in each solution. In hypertonic conditions, the cells are visibly plasmolyzed as evident from optical microscopy images after the first minute of exposure to sorbitol (See Figure A4). We note that the plasmolysis is evident for cells in stages 0–2 when the cytoplasm is still inside the cells. In isotonic and hypotonic conditions, the cell physiology as studied by optical microscopy is the same as in their normal growth conditions (cells are turgid, cytoplasm pushing against the cell wall). While we refer to cells treated with growth media as being in an isotonic condition, they are turgid, and the turgor pressure drives their growth and development. We use the term isotonic in a relative sense, as compared to the high osmotic pressure differential in hypotonic conditions. In the case of deionized water-suspended cells, the higher osmotic pressure (see Table A3) causes higher stress on the cell walls [30]. In Figure 4 the initial effective stiffness values for the overall cell in each stage of differentiation, are presented grouped by osmolarity of solution. Underneath each category is a graphical illustration of the morphology of the cells. The initial effective stiffness is measured from the first 1 μm of indentation data after contact. This depth of indentation ensures that we capture some contribution to the overall stiffness from turgor pressure and/or the cytoplasm, since the measured (hydrated) PCW and SCW thicknesses are both close to 1 μm [11]. See Figure A5 for an alternative grouping of the stiffness measurements by stage and osmolarity of solution.

#### 2.2.1. Hypertonic Condition

In hypertonic conditions, i.e., the sorbitol condition (Figure 4A), water flows out of the vacuole and across the cell membrane, as the cell is plasmolyzed. The relief of turgor pressure allows for isolation of the mechanical response of the cell wall [9,31]. So, in these conditions we model the stiffness response of the cell as a single spring which represents the cell wall(s). When uninduced cells (stage 0) are placed in hypertonic conditions, the measured stiffness ($k_{\mathrm{hyper},\mathrm{stage}\;0}=0.59\pm 0.06\;N/m$) corresponds to that of unstressed PCW.

(2) $$k_{\mathrm{hyper},\mathrm{stage}\;0}=k_{\mathrm{hyper},\mathrm{PCW}}$$

After DEX exposure, but before the formation of the SCW begins, we do not expect to see a difference in the stiffness of the PCW. Indeed, we do not detect any statistically significant difference in stiffness between stages 0 and 1 in hypertonic conditions ($k_{\mathrm{hyper},\mathrm{stage}\;1}=0.69\pm 0.17\;N/m$).

(3) $$k_{\mathrm{hyper},\mathrm{stage}\;1}=k_{\mathrm{hyper},\mathrm{PCW}}$$

In stage 2, the PCW is expanded and modified to allow space for the deposition of the SCW [25]. The coupled stiffness of the thin SCW bundles and the modified PCW interact in a way that produces a significant increase on the cellular effective stiffness ($k_{\mathrm{hyper},\mathrm{stage}\;2}=4.71\pm 2.31\;N/m$); this value is over five times the value observed in the prior stages, and more than double the addition of the isolated PCW stiffness and isolated SCW stiffness from stages 0 and 3. We propose two possible reasons for the observed increase in stiffness.

The presence of sorbitol may cause an increase of the cell wall stiffness through enhanced molecular interactions between the polysaccharide chains of the PCW and SCW. We hypothesize that in the presence of sorbitol, a polyalcohol with six hydroxyl groups per molecule, these side groups can interact with the available surface hydroxyl groups of the various polysaccharide chains (i.e., cellulose, hemicellulose, pectin) in the PCW and SCW. These polysaccharides are present on each cell wall in different amounts and configurations, and the interactions between them are a topic of active investigations [32]. The introduction of sorbitol may therefore contribute additional hydrogen bonding between the PCW and SCW, supporting our micro-indentation experimental observations.

Alternatively, the collapse of the cell in hypertonic conditions may cause buckling or folding of the PCW over the SCW bundles. Cell wall buckling or folding would result in more amount of cell wall material being compressed under the indenter, thereby justifying a higher stiffness. This apparent stiffening in plasmolyzing conditions due to cell wall buckling has been suggested also for plant tissue indentations [31]. Again, this phenomenon would exist in all stages, but would be enhanced when the PCW is in contact with the spiral SCW. The gaps between the spiral SCW bundles provide channels in between which the PCW could fold, giving the overall cell wall material a thicker and more organized shape.

These two proposed mechanisms for stiffening are not mutually exclusive. The sorbitol may be interacting with cellulose in the PCW as it buckles to provide an even further increase in stiffness for the reorganized overall cell wall structure. In any case, the combined cell walls (CCWs) are the material which provides stiffness to the cell in the hypertonic condition.

(4) $$k_{\mathrm{hyper},\mathrm{stage}\;2}=k_{\mathrm{hyper},\mathrm{CCW}}$$

As PCD proceeds, all contents of the cytoplasm are lost and the PCW is at least partially hydrolyzed. At the end of transdifferentiation, the main remaining structural component of the xylem vessel element is a thickened SCW. In the final stage, in all solutions, we attribute all the measured stiffness to the thickened SCW ($k_{\mathrm{hyper},\mathrm{stage}\;3}=1.03\pm 0.13\;N/m$).

(5) $$k_{\mathrm{hyper},\mathrm{stage}\;3}=k_{\mathrm{SCW}}$$

We measure a statistically significant higher stiffness in stage 3 when compared to stages 0 and 1 in hypertonic conditions. This result suggests that the fully developed SCW is stiffer than the PCW before and after induction. We also measure a statistically significant lower stiffness in stage 3 when compared to stage 2 in hypertonic conditions. The loss of the PCW as PCD proceeds eliminates the stiffening that was observed in stage 2, through either (or both) chemical and physical interactions explained in the paragraphs above.

#### 2.2.2. Isotonic Condition

In isotonic conditions (Figure 4B), the cells are growing, so the turgor pressure is above the osmotic pressure of the solution, but not as high as in the hypotonic condition discussed in the next section (see Table A3). In growth media the cells are turgid, the cytoplasm pushes against the cell wall, presenting a distinct morphological difference compared to the plasmolyzed cells which are shriveled and have a retracted cytoplasm. As shown in literature, turgor pressure (from the vacuole and the cytoplasm) provide additional mechanical stiffness to the cell underneath the cell wall [11,33,34]. They are represented by a new spring in our model connected in series to the cell wall(s). Before PCD is initiated, in stages 0 and 1, due to the presence of turgor pressure, we expect the stiffness of the PCW to be higher than in the same stages in hypertonic conditions because it is stressed ($k_{\mathrm{iso},\mathrm{PCW}}>k_{\mathrm{hyper},\mathrm{PCW}}$).

In stage 0, before transdifferentiation is initiated, we observe the lowest stiffness among the cells tested in isotonic solution ($k_{\mathrm{iso},\mathrm{stage}\;0}=0.82\pm 0.52\;N/m$). From our spring model, the overall stiffness of the cell in isotonic conditions in stage 0 is

(6) $$k_{\mathrm{iso},\mathrm{stage}\;0}=\frac{k_{\mathrm{iso},\mathrm{PCW}}\left(k_{n-b,\mathrm{iso}}\right)}{k_{\mathrm{iso},\mathrm{PCW}}+k_{n-b,\mathrm{iso}}}$$

There is no statistically significant difference between the effective stiffness of cells in stage 0 in hypertonic and isotonic conditions. For the effective spring constants in both of these models to be equivalent, the two springs in series in the isotonic model must be stiffer than the single spring in the hypertonic model (see plot in Figure 3). This implies that the PCW and the combined cytoplasm and vacuole in isotonic conditions must be stiffer than the PCW in hypertonic conditions. In other words, our model confirms that the PCW is stiffened through stress exerted from turgor pressure that exists in isotonic conditions.

Upon induction of transdifferentiation, the effective stiffness of the cells increases significantly. In stage 1, the model still contains 2 springs: one for the stressed PCW ($k_{\mathrm{iso},\mathrm{PCW}}$), and one for the bundled cytoplasm in isotonic conditions ($k_{b,\mathrm{iso}}$). The mean effective stiffness in stage 1 is $k_{\mathrm{iso},\mathrm{stage}\;1}=2.40\pm 0.52$ N/m.

(7) $$k_{\mathrm{iso},\mathrm{stage}\;1}=\frac{k_{\mathrm{iso},\mathrm{PCW}}\left(k_{b,\mathrm{iso}}\right)}{k_{\mathrm{iso},\mathrm{PCW}}+k_{b,\mathrm{iso}}}$$

Assuming that there is no change in the PCW stiffness from stages 0 to 1, as observed in sorbitol (Figure 4A), our model indicates that the cytoplasmic contribution in stage 0 must be less than in stage 1 in isotonic conditions ($k_{n-b,\mathrm{iso}}<k_{b,\mathrm{iso}}$).

As transdifferentiation proceeds to stage 2, a series of concurrent events influence the mechanical behavior of the cells: (i) the PCW is modified (loosened to allow for elongation for the SCW deposition and possibly entering the hydrolysis stage) (ii) the beginnings of SCW bundles are deposited, and (iii) the anisotropic fibrillar cytoplasm starts detaching from the cell walls as the turgor pressure is reduced as a result of the cell entering the PCD stage upon differentiation [25]. The stiffness of the new CCW is represented in the spring model as $k_{\mathrm{iso},\mathrm{CCW}}$, and the spring from the cytoplasm is removed, since the cytoplasm is no longer in contact with the cell wall. These mechanisms act together to determine the effective stiffness of the cell ($k_{\mathrm{iso},\mathrm{stage}\;2}=2.06\pm 0.44\;N/m$). The reduced turgor pressure, loss of cytoplasmic contribution as the cell dies and the PCW loosening reduce the effective stiffness of the system. The deposition of SCW increases the stiffness of the cell wall spring component, and therefore the overall system. According to our experiments, the cells have the same stiffness in stages 1 and 2. Thus, assuming that the cytoplasmic contribution is negligible at stage 2, the stressed PCW of stage 1 must be stiffer than the CCW of stage 2 ($k_{\mathrm{iso},\mathrm{CCW}}<k_{\mathrm{iso},\mathrm{PCW}}$). This highlights the significant effects of turgor pressure stiffening the PCW in stages 0 and 1.

(8) $$k_{\mathrm{iso},\mathrm{stage}\;2}=k_{\mathrm{iso},\mathrm{CCW}}$$

At the last stage of differentiation when the SCW is fully developed and thickened, the measured effective stiffness ($k_{\mathrm{iso},\mathrm{stage}\;3}=1.78\pm 0.97\;N/m$) is not statistically significant from the overall stiffnesses in stages 1 and 2. In order for cells in stage 3 to have the same overall stiffness as cells in stage 1, each component of the springs in series in stage 1 must be stiffer than the single spring in stage 3 ($k_{\mathrm{SCW}}<k_{\mathrm{iso},\mathrm{PCW}}$). This highlights again the increased stiffness of a PCW under turgor pressure in stages 0 and 1.

(9) $$k_{\mathrm{iso},\mathrm{stage}\;3}=k_{\mathrm{SCW}}$$

#### 2.2.3. Hypotonic Condition

In water (Figure 4C), before the DEX induction, we measure the absolute stiffest cell response ($k_{\mathrm{hypo},\mathrm{stage}\;0}=7.37\pm 1.58$ N/m). This demonstrates that in hypotonic conditions, the high turgor pressure increases the stiffness of cytoplasm and the PCW. This is in agreement with the findings of Routier-Kierzkowska et al. and many others who have studied the effects of turgor pressure on cell and tissue mechanics [12,33,35,36,37].

(10) $$k_{\mathrm{hypo},\mathrm{stage}\;0}=\frac{k_{\mathrm{hypo},\mathrm{PCW}}\left(k_{n-b,\mathrm{hypo}}\right)}{k_{\mathrm{hypo},\mathrm{PCW}}+k_{n-b,\mathrm{hypo}}}$$

According to the two-spring model, this means that both the stiffness of the PCW and the cytoplasm in hypotonic conditions in stage 0 must be greater than any other directly measured stiffness.

As differentiation begins, the overall stiffness of the cell ($k_{\mathrm{hypo},\mathrm{stage}\;1}=1.89\pm 0.48$ N/m) is drastically reduced.

(11) $$k_{\mathrm{hypo},\mathrm{stage}\;1}=\frac{k_{\mathrm{hypo}*,\mathrm{PCW}}\left(k_{b,\mathrm{hypo}}\right)}{k_{\mathrm{hypo}*,\mathrm{PCW}}+k_{b,\mathrm{hypo}}}$$

Assuming that there is no decrease in the cytoplasmic stiffness from stages 0 to 1, there must be lower stress in the PCW in stage 1, which we will denote $k_{\mathrm{hypo}*,\mathrm{PCW}}$. We hypothesize that the stress exerted on the cell wall is reduced as the cell prepares for SCW deposition ($k_{\mathrm{hypo},\mathrm{PCW}}>k_{\mathrm{hypo}*,\mathrm{PCW}}$). Loosening of the PCW to prepare for elongation prior to addition of PCW material has been previously reported, and here we propose that this same mechanism governs SCW deposition [4]. This loosening should be occurring in all osmotic conditions, but we propose that it is only distinguishable in hypotonic conditions because in these conditions the PCW is under the highest amount of stress since it is subjected to the highest turgor pressure. An alternative, or additional possible mechanism for this observed weakening is an early hydrolysis of the PCW. Both of these possibilities are supported by literature [25]. Our analysis could not distinguish the stiffness of the PCW in water stage 1 ($k_{\mathrm{hypo}*,\mathrm{PCW}}$) from the stiffness of the PCW in growth medium at the same stage ($k_{\mathrm{iso},\mathrm{PCW}}$).

As SCW is deposited, we measure that the effective stiffness at stage 2 is the lowest among all stages in hypotonic treatment ($k_{\mathrm{hypo},\mathrm{stage}\;2}=0.71\pm 0.14$ N/m).

(12) $$k_{\mathrm{hypo},\mathrm{stage}\;2}=k_{\mathrm{hypo},\mathrm{CCW}}$$

As seen before, the balance between PCW modification (loosening/hydrolysis), early SCW deposition, and loss of turgor and cytoplasmic contribution determines the overall system stiffness. The effective stiffness of cells at stage 2 in hypotonic conditions is significantly lower than that of cells at stage 2 in isotonic and hypertonic conditions (see Figure A5). This difference in stiffness between water and other solutions in stage 2 supports the prior proposal that there is weakening of PCW due to early hydrolysis in water.

Finally in the last stage the measured stiffness ($k_{\mathrm{hypo},\mathrm{stage}\;3}=1.12\pm 0.15$ N/m) corresponds solely to the fully developed SCW.

(13) $$k_{\mathrm{hypo},\mathrm{stage}\;3}=k_{\mathrm{SCW}}$$

Our results indicate that the combined CW stiffness of stage 2 is weaker than the mature SCW ($k_{\mathrm{hypo},\mathrm{CCW}}<k_{\mathrm{SCW}}$). As expected, we measure that the thickened SCW in any solution has the same stiffness, which shows that the properties of the fully developed SCW are not affected by the treatments. We have two cases of exceptionally high stiffness; uninduced cells in hypotonic conditions and plasmolyzed cells in stage 2 of transdifferentiation. Besides these two exceptional cases, the SCW alone is at least as stiff as any combined stiffnesses in any other case.

To summarize, the isolated CW stiffnesses can be ordered:

(14) $$k_{\mathrm{PCW}}<k_{\mathrm{SCW}}<k_{\mathrm{iso},\mathrm{PCW}},k_{\mathrm{hypo}*,\mathrm{PCW}}<k_{\mathrm{hypo},\mathrm{PCW}}$$

Again, we see that turgor pressure governs the overall mechanical response of the cell to compression through prestressing the PCW. We also confirm that the SCW bundles are stiffer than the PCW material without any prestress.

The CCW stiffnesses can be ordered as follows:

(15) $$k_{\mathrm{hypo},\mathrm{CCW}}<k_{\mathrm{iso},\mathrm{CCW}}<k_{\mathrm{hyper},\mathrm{CCW}}$$

The proposed molecular mechanisms governing the stiffness of the CCW are the hydrolysis of the PCW in water, and the stiffening of cellulose chains in the presence of sorbitol. Buckling or folding of the PCW in hypertonic conditions may also act to further stiffen the CCW response.

Finally, the stiffness representing the cytoplasm can be constrained with two inequalities:

(16) $$k_{n-b,\mathrm{iso}}<k_{b,\mathrm{iso}}<k_{n-b,\mathrm{hypo}}$$

(17) $$k_{n-b,\mathrm{iso}}<k_{b,\mathrm{hypo}}$$

**Figure A6:**
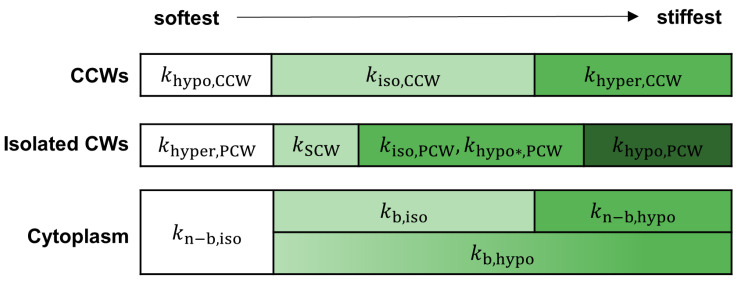
Stiffness comparison for each component of spring models.

See Figure A6 for a visual representation of the magnitude of each stiffness component. Our assay allows us to directly assess, for the first time, the mechanical contributions of the cytoskeleton in the effective stiffness of intact plant cells, highlighting their important role in the mechanics of the system.

### 2.3. AFM Analysis of Differentiating VND7-Inducible Arabidopsis Cells

**Figure 5:**
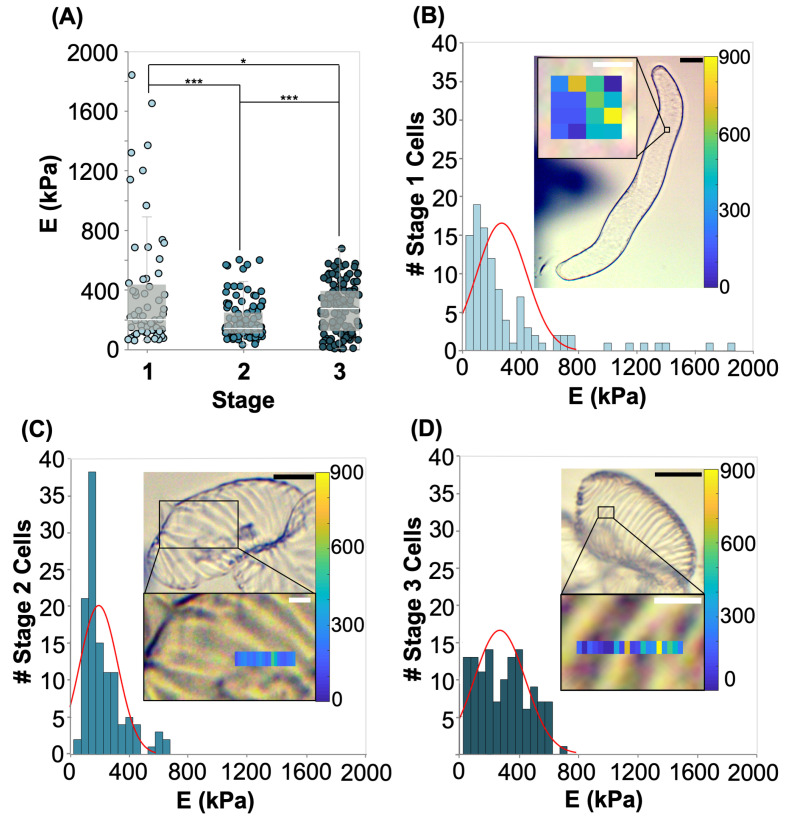
(**A**) Young’s modulus for differentiating VND7-inducible Arabidopsis cells in each stage of differentiation measured with AFM. Stars indicate significant differences in distribution according to a Kolmogorov-Smirnov test. * *p* < 0.1, *** *p* < 0.01. (Data shown correspond to n > 60. Each nano-indentation test is represented by a point in the plot. A minimum of 7 individual cells were tested in each stage.) (**B**) Histogram of Young’s moduli measured in stage 1 of differentiation. Inset shows example location of measurement and map of stiffness in the area. (**C**) Histogram of Young’s moduli measured in stage 2 of differentiation. Inset shows example location of measurement on cell and line map of stiffness in the area. (**D**) Histogram of Young’s moduli measured in stage 3 of differentiation. Inset shows example location of measurement on cell and line map of stiffness in the area. Inset image scale bars are 20 μm (black). Zoomed-in inset scale bars are 2 μm (white).

AFM nano-indentation tests were conducted in water to evaluate cell wall indentation moduli in each stage of differentiation, as shown in Figure 5. We use a spherical bead with a 1 μm diameter, which is able to capture the response of a rather large representative area of the PCW, considering the fact that cellulose fibrils are organized in bundles with 5–50 nm thickness [3]. The average indentation depth for the force-controlled experiment is 79.5±3.9 nm (Mean ± Standard Error), which is less than 10% of the average thickness of the hydrated PCW or SCW bundles. Therefore, the indentation depth is adequately shallow to assume that we can isolate the response of the cell wall, even though the cells are turgid [11,14,15,31]. Young’s moduli measured from the PCW in stage 1 in hypotonic conditions ($E_{\mathrm{hypo},\mathrm{stage}\;1}=372\pm 51$ kPa) is higher than in other stages of differentiation, which is in agreement with our micro-indentation results. The Young’s moduli measured from the CCW in stage 2 ($E_{\mathrm{hypo},\mathrm{stage}\;2}=192\pm 13$ kPa) is the lowest of the three stages, again confirming our measurements from the micro-indentation test. Finally, the Young’s moduli measured in stage 3 ($E_{\mathrm{hypo},\mathrm{stage}\;3}=271\pm 15$ kPa) has an intermediate stiffness, which further validates our micro-indentation results.

Measurements with AFM illustrate the extremely heterogeneous structure of the CW. In stage 1, where the PCW is the only CW of the system, the indentation modulus is measured in a range of 58.7 to 1840 kPa as shown in the histogram and map inset of Figure 5B. This large distribution arises from the heterogeneous, fibrillar structure of the PCW. The distribution of rigid cellulose fibrils in the compliant heterogeneous matrix of polysaccharides, proteins and phenolic compounds, is causing the local distribution of stiffness we observe with AFM. The high stress in the PCW in hypotonic solution leads to a high stress in the fibres of the PCW, which amplifies the observed heterogeneous stress distribution. In stage 2, the indentation modulus is measured in a range of 31.0 to 601 kPa (Figure 5C). The higher number of measurements with low moduli in stage 2 illustrate the degradation of the PCW, especially between SCW bundles, which was also suggested from the micro-compression tests. The overlay of line scan measurements on images of the cell reveals that we observe the higher moduli when testing over the combined early SCW bundles and PCW. In the example shown as an inset in Figure 5C, we see a modulus of approximately 600 kPa over the SCW bundle, and moduli around 300 kPa between the bundles. In stage 3, the indentation modulus is measured in a range of 5.6 to 676 kPa. The inset of Figure 5D shows a line scan over an area containing two SCW bundles. The line scan shows that the moduli on top of the bundles is as high as 700 kPa, and between the bundles they are about 150 kPa. The indentation moduli measured in stage 3 are more uniformly distributed between the minimum and maximum values than in stage 2. As the SCW bundles thicken, they become stiffer and eliminate the intermittent spaces, leading to fewer measurements over only degraded PCW.

## 3. Materials and Methods

### 3.1. Cell Culture and Differentiation Induction

A suspension culture of transgenic *Arabidopsis thaliana* cells (VND7-inducible line, VND7-VP16-GR) was prepared from T87 cell line as described by [16]. The cells were maintained as callus form by the culture on solid agar medium, and transferred to new medium every 3 weeks. Parts of the callus of VND7-inducible cells were used to initiate a suspension culture, which was passaged weekly and was kept in flasks on a rotary shaker at 130 rpm at 23 ${}^{\circ }$C. The VND7-inducible cell suspension was maintained in a modified Murashige and Skoog (MS) medium (Duchefa, Haarlem, The Netherlands) supplemented with 87 mM sucrose, 1 nM 2,4-dichlorphenoxyacetic acid, 555 nM myo-inositol, 2 nm thiamin, 34 nM kanamycin, and 1.5 mM Potassium phosphate. To induce differentiation, dexamethasone (DEX) was introduced to the liquid media at a final concentration of 10 μM. Cells were collected post induction from the cultures at different time points and their stage was classified from their morphological features as mentioned in Section 2.1. All chemicals and reactants were purchased from MilliporeSigma (St. Louis, MO, USA).

### 3.2. Microscopy Observations

Cell walls were stained with 0.005% (*w/v*) calcofluor white and observed under a laser scanning confocal microscope (LSM880, Zeiss, Oberkochen, Germany). Cells were extracted from the culture, immersed in staining solution and imaged without any other treatment. Z-stacks were acquired using a 40× water immersion objective (NA 1.2) and Imaris 9.5 (Bitplane, Zurich, Switzerland) was used for 3D rendering and bundle width determination. Specifically the Imaris Measurement Points module was used to quantify the bundles and the Surface module was used to reconstruct the PCW and SCW.

For light microscopy observations, which were performed to measure the dimensions of the cells, the cell walls of freshly extracted cells from culture were stained with 1 vol% solution of alcian blue in 3 vol% acetic acid, and observed with an AxioScope A1 (Zeiss, Oberkochen, Germany). Image analysis was carried out in ImageJ (http://rsb.info.nih.gov/ij/). From individual length measurements we report the statistical mean and standard error in the main part of the manuscript.

### 3.3. Mechanical Testing

We tested the mechanical properties of the cells in three different osmotic conditions: in pure deionized water, in 1M sorbitol, and in growth media (composition mentioned above).

**Figure A7:**
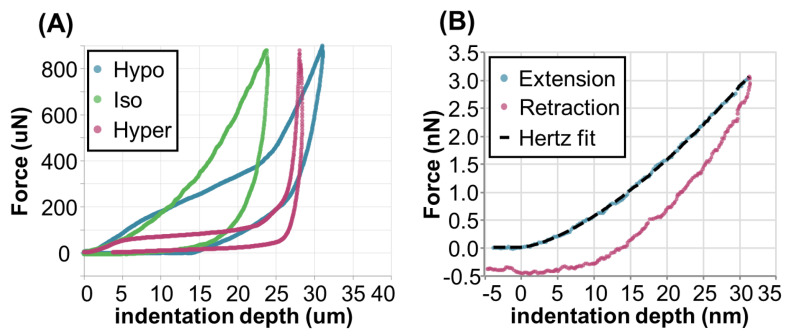
Example force-indentation data for (**A**) micro-compression experiment on three different cells in each of the three osmotic treatments; hypotonic, isotonic, and hypertonic, and (**B**) AFM experiment with extension (blue line) and retraction (pink line) data with Hertz fit (dash line) overlay.

The micro-compression tests were performed using a FT-MTA02 system equipped with FT-S1000-LAT (liquid design) sensing probes with a 50 × 50 $\mu m^{2}$ square tip (FemtoTools AG, Zurich, Switzerland) and an optical microscope. The obtained data of the indentations were position-corrected to account for contributions of the system’s stiffness. Microscope glass slides (AmScope, Irvine, CA, USA) were cleaned with isopropyl alcohol, surface activated with a high frequency generator for 1 min (BD-20A, Electro-Technic Products, Chicago, IL, USA), and a thin layer of 0.5 mL of poly-l-lysine was spin coated on top of the slides (SUSS MicroTec, Garching, Germany). Cells were extracted from culture and pipetted on the coated glass slides. The cells were washed several times with the selected treatment solution to effectively decluster them and keep only the ones that adhered better to the substrate. For testing 1–3 mL of the selected solution were added on top of the washed and diluted cells, and force-controlled indentations to 900 μN were conducted by immersing the sensing probe in liquid. The corresponding average indentation depth was 29.2±0.93μm (Mean ± Standard Error), which is close to the average width of the cell reported in Table A1 because we compress most cells to failure. In the plots on Figure 4 each point corresponds to the compression of an individual cell. Representative force indentation curves of cells in stage 0 in all tested osmotic conditions are presented in Figure A7A.

Short-range nano-indentations to evaluate the properties of the cell wall were conducted with AFM (Asylum Research, MFP-3D-Bio, Goleta, CA, USA). For the indentations, we used custom tips with a silicon dioxide spherical particle (diameter 1 μm) on a silicon nitride (SN) cantilever with a stiffness of 0.63±0.03 N/m (Mean ± Standard Error) and a virtual deflection of 14.9±4.5 mV/μm (Mean ± Standard Error) (Novascan, Boone, IA, USA). The AFM indentations were conducted in dionized water, in glass slides treated as mentioned before for the micro-compression tests. For every tested glass slide the system was allowed to reach thermal equilibrium for 2–3 h. We conducted force-controlled indentations to 3nN and applied the Hertz model to calculate the indentation modulus, E. Each point in Figure 5A corresponds to an indentation test. We conducted multiple indentations for a given cell, and tested a minimum of 7 cells in each stage. A representative force indentation curve in which the indentation part has been fitted with a Hertz model is presented in Figure A7B.

### 3.4. Analysis

Most of the data processing for the micro-compression tests follows that of Routier-Kierzkowska et al. [12]. First, to account for the compliance of the sensor, a reference measurement is obtained by compressing an area of the glass slide with no cells present for 1–2 μm. The linear indentation part of data are linearly fitted. The sensor stiffness (*S*) is typically above 200 N/m. All data sets are then transformed by

(18) $$\delta_{\mathrm{corrected}}=\delta -\frac{F}{S}$$

where $\delta_{\mathrm{corrected}}$ is the corrected displacement, δ is the measured displacement, *F* is the measured force, and *S* is the sensor stiffness determined by calibration.

Next, we offset the measured force-displacement data so that the average force up until the contact point is zero. The contact point is defined as the point where the force exceeds a user-defined threshold. The force thresholding and offsetting are repeated using increasingly sensitive force thresholds. The final selected threshold value is typically less than 1 μm. Then, a Savitsky-Golay moving-window data filter is applied to smooth the data. The window size is 25 data points which are fit to a 2nd order polynomial.

Finally, the first 1 μm of indentation data after the located contact point are linearly fitted. The interpolated slope is taken as the overall stiffness of the cell. The overall stiffnesses of cells are compared between stages of transdifferentiation and between osmolarities of testing solutions. A Kolmogorov-Smirnov statistical test is performed which compares the empirical cumulative distribution functions of each grouping.

The data processing for AFM nano-indentations was executed by Asylum Research software (AR 16.10.211) in Igor Pro 6.3. The software was used to identify the contact point and extract a Young’s modulus through the application of the Hertz model.

All data processing was performed using the Python programming language (Python Software Foundation, https://www.python.org/). All statistical visualizations were created using Altair [38].

## 4. Conclusions

We designed a multi-scale biomechanical assay to experimentally isolate the mechanical contributions from the cytoplasm and cell wall during the differentiation of transgenic Arabidopsis cells to protoxylem vessel elements. The mechanical data at different scales and in different osmotic conditions in combination with the proposed two spring model, allow us to decouple the contributions from each structural element of the cell as it responds to changes in turgor pressure at various stages of the differentiation process. At the micro-scale, we performed indentations that covered most of the cell area and measured the overall stiffness from the first 1 μm of indentation data, ensuring that we probe stiffness contributions from the cell wall and cytoplasm, which we represent as two springs in series in our proposed spring model. The resulting deconvoluted stiffnesses from the cell wall(s) and cytoplasm are dependent on the validity of the assumptions required to implement the spring model, which includes neglecting any nonlinear mechanical effects, like adhesion, viscosity or plasticity. At the nano-scale, we performed AFM indentations that covered a smaller area of the cell wall, and indented a few tens of nanometers, to isolate the mechanical behavior of the cell wall. Our analysis provides experimental evidence that the SCW is stiffer than the relaxed PCW, in a living cell system. This conclusion is reached by comparing measured cell stiffnesses in hypertonic conditions, where the cell wall is effectively decoupled from the cytoplasm. In isotonic and hypotonic conditions, turgor pressure gives rise to an increased stress in PCW, causing it to stiffen beyond the SCW. We also measure a quantifiable loosening of the PCW in stage 1 in hypotonic conditions, as the cell prepares for deposition of the SCW. This is the first time a mechanical weakening is measured on the PCW before the SCW deposition in living cells. From measurements in isotonic and hypotonic conditions, we also find evidence of a quantifiable difference in cytoplasmic stiffness as a consequence of active bundling of the filaments in the cytoplasm, guided by differentiation.

These findings provide insight into the mechanisms of xylem vessel element differentiation. They suggest that inter- and/or intra-cellular mechanical signals regulate cell differentiation and SCW deposition.

## Figures and Tables

**Table 1:** Measured principal dimensions and feature sizes of elongated VND7-inducible cells in the 3 stages of transdifferentiation. Length (*L*), width (*w*), and thickness (*t*) of SCW bundles are measured using image processing. Volume (*V*) is calculated from measured length and width, assuming cells are cylindrical in shape. The density of SCW bundles (ρ) is calculated by counting the number of bundles observed in a particular cell, and dividing by the cross-sectional area in the image.

Dimension	Mean ± SE
$L_{\mathrm{stage}}\;{}_{1}$ (μm)	60.4±2.4
$L_{\mathrm{stage}}\;{}_{2}$ (μm)	56.4±4.9
$L_{\mathrm{stage}}\;{}_{3}$ (μm)	61.6±3.7
$w_{\mathrm{stage}}\;{}_{1}$ (μm)	30.7±1.0
$w_{\mathrm{stage}}\;{}_{2}$ (μm)	31.2±2.8
$w_{\mathrm{stage}}\;{}_{3}$ (μm)	34.7±1.6
$V_{\mathrm{stage}}\;{}_{1}$ ($\mu m^{3}$)	44,700±2100
$V_{\mathrm{stage}}\;{}_{2}$ ($\mu m^{{}_{3}}$)	43,100±5300
$V_{\mathrm{stage}}\;{}_{3}$ ($\mu m^{{}_{3}}$)	58,300±4100
$\rho_{\mathrm{stage}}\;{}_{2}$ ($\#/\mu m^{2}$)	0.056±0.005
$\rho_{\mathrm{stage}}\;{}_{3}$ ($\#/\mu m^{2}$)	0.060±0.004
$t_{\mathrm{stage}}\;{}_{2}$ (μm)	1.05±0.01
$t_{\mathrm{stage}}\;{}_{3}$ (μm)	1.45±0.01

## Abbreviations

The following abbreviations are used in this manuscript:

AFM	Atomic-force microscopy
DEX	dexamethasone
$k_{\mathrm{hypo}/\mathrm{iso}/\mathrm{hyper},\mathrm{stage}\;0/1/2/3}$	overall stiffness of cell in hypotonic/isotonic/hypertonic solution in stage 0/1/2/3
$k_{\mathrm{hypo}/\mathrm{iso}/\mathrm{hyper},\mathrm{PCW}}$	stiffness of primary cell wall in hypotonic/isotonic/hypertonic solution
$k_{\mathrm{hypo}*,\mathrm{PCW}}$	stiffness of primary cell wall in hypotonic solution in stage 1, which is
	somehow weakened
$k_{\mathrm{hypo}/\mathrm{iso}/\mathrm{hyper},\mathrm{CCW}}$	stiffness of combined cell walls in hypotonic/isotonic/hypertonic solution
$k_{\mathrm{SCW}}$	stiffness of secondary cell wall
$k_{n-b,\mathrm{hypo}/\mathrm{iso}}$	stiffness of non-bundled cytoplasm in hypotonic/isotonic solution
$k_{b,\mathrm{hypo}/\mathrm{iso}}$	stiffness of bundled cytoplasm in hypotonic/isotonic solution
$E_{\mathrm{hypo},\mathrm{stage}\;1/2/3}$	Young’s modulus from AFM indentations on cells in hypotonic solution in
	stage 1/2/3
PCW	Primary cell wall
CCW	combined cell walls
SCW	Secondary cell wall
TE	Tracheary element
PCD	Programmed cell death
SE	Standard error
VND7	VASCULAR-RELATED NAC-DOMAIN7
MT	Microtubule

## Appendix A

The mean principal dimensions and standard errors for each cell shape (rounded and elongated) are reported in Table A1. The confocal analysis reveals that in stage 1, the PCW thickness of rounded and elongated cells is the same, measured at 580±10 nm (Mean ± Standard Error) in both cases. The population of rounded cells that have transitioned into stage 2 and have deposited SCW bundles, have on average a total cell volume 72% higher than rounded cells at stage 1. Cells that are found to be in stage 3, with fully developed SCW and ruptured tonoplast, have on average an insignificantly changed total volume, compared to cells at stage 2. Our observations indicate a different trend in elongated cells. In that case, the volume of cells in stages 1 and 2 is almost the same. Between elongated cells in stages 2 and 3, we notice that cells at the last stage of differentiation are about 35% on average larger in volume. The higher volume of cells in stage 3, reflects larger dimensions in both the lateral (11% average increase in width) and longitudinal directions (9% average increase in length). Comparing the volumes of the two shapes, elongated cells are approximately 69% larger than rounded cells in stage 1. In stage 2, elongated and rounded cells have similar volumes. Finally, in stage 3, elongated cells have 22% more volume than rounded cells on average. Apparently, rounded cells are able to enlarge more significantly than elongated cells just prior to SCW deposition.

**Table A1:** Principal dimensions of elongated and round shaped cells. Length, width and diameter were measured from light microscopy images. Volumes were calculated using formula for a cylinder for elongated cells and a sphere for round cells. The data shown correspond to Mean ± Standard Error (SE) (n > 20).

Cell Shape & Stage	Dimension	Mean ± SE
Elongated Stage 1	Length (μm)	60.4±2.4
Elongated Stage 2	Length (μm)	56.4±4.9
Elongated Stage 3	Length (μm)	61.6±3.7
Elongated Stage 1	Width (μm)	30.7±1.0
Elongated Stage 2	Width (μm)	31.2±2.8
Elongated Stage 3	Width (μm)	34.7±1.6
Round Stage 1	Diameter (μm)	37.0±0.9
Round Stage 2	Diameter (μm)	44.2±2.1
Round Stage 3	Diameter (μm)	45.0±2.0
Elongated Stage 1	Volume ($\mu m^{3}$)	44,700 ± 2100
Elongated Stage 2	Volume ($\mu m^{3}$)	43,100±5300
Elongated Stage 3	Volume ($\mu m^{3}$)	58,300±4100
Round Stage 1	Volume ($\mu m^{3}$)	26,500±1000
Round Stage 2	Volume ($\mu m^{3}$)	45,500±3300
Round Stage 3	Volume ($\mu m^{3}$)	47,700±3400

In Table A2 we present the thickness and areal densities of SCW bundles in rounded and elongated cells. We measure that the elongated and rounded cells have similar bundle densities and thicknesses in stage 2. We observe that the SCW thickening during the last stage of differentiation leads to the same bundle thickness in both elongated and rounded cells. In both cases the SCW thickens by approximately 40%, as presented in Table A2. The bundle density does not change significantly between stages 2 and 3 for either elongated or rounded cells. The measured bundle density is about 7% higher for elongated cells than for rounded cells. We propose that this is a result of the inherent structural requirement of elongated vessels to be able to support higher stresses in their walls than spherical vessels when experiencing the same amount of pressure.

**Table A2:** SCW bundle feature sizes for rounded and elongated VND7-inducible cells. Bundle densities were measured from optical microscopy images. Bundle thicknesses were measured from confocal three-dimensional reconstructions. The data shown correspond to Mean ± Standard Error (SE) (n > 45).

Cell Shape & Stage	Dimension (μm)	Mean ± SE
Elongated Stage 2	bundle density ($\#/\mu m^{2}$)	0.056±0.005
Elongated Stage 3	bundle density ($\#/\mu m^{2}$)	0.060±0.004
Round Stage 2	bundle density ($\#/\mu m^{2}$)	0.051±0.005
Round Stage 3	bundle density ($\#/\mu m^{2}$)	0.050±0.008
Elongated Stage 2	bundle thickness (μm)	1.05±0.01
Elongated Stage 3	bundle thickness (μm)	1.45±0.01
Round Stage 2	bundle thickness (μm)	1.09±0.04
Round Stage 3	bundle thickness (μm)	1.52±0.03

The relationship between turgor pressure and osmolarity is described by the following equation

(A1) P=MiRT

where *P* is the osmotic pressure, *M* is the molarity of the solution, *i* is the Van’t Hoff factor which represents the number of distinct particles produced when the substance is dissolved, *R* is the universal gas constant, and *T* is temperature [30].

**Table A3:** Osmotic pressure calculated for each of the three solutions used for mechanical testing.

Solution	*P* (MPa)
water	137.5
T87 Growth Medium	11.4
sorbitol	2.5
